# Bioprotective Effect of *Lactococcus piscium* CNCM I-4031 Against *Listeria monocytogenes* Growth and Virulence

**DOI:** 10.3389/fmicb.2018.01564

**Published:** 2018-07-17

**Authors:** Taous Saraoui, Françoise Leroi, Frédérique Chevalier, Jean-Michel Cappelier, Delphine Passerini, Marie-France Pilet

**Affiliations:** ^1^Laboratoire Ecosystèmes Microbiens et Molécules Marines pour les Biotechnologies (EM^3^B), L’Institut Français de Recherche pour l’Exploitation de la Mer (Ifremer), Nantes, France; ^2^UMR1014 SECALIM, INRA, École Nationale Vétérinaire, Agroalimentaire et de l’Alimentation de Nantes-Atlantique (ONIRIS), Nantes, France

**Keywords:** biopreservation, *Lactococcus piscium*, *Listeria monocytogenes*, co-culture, cell ratio, scanning electron microscopy, virulence

## Abstract

*Listeria monocytogenes* is a Gram-positive pathogen occurring in many refrigerated ready-to-eat foods. It is responsible for foodborne listeriosis, a rare but severe disease with a high mortality rate (20–30%). *Lactococcus piscium* CNCM I-4031 has the capacity to prevent the growth of *L. monocytogenes* in contaminated peeled and cooked shrimp and in a chemically defined medium using a cell-to-cell contact-dependent mechanism. To characterize this inhibition further, the effect of *L. piscium* was tested on a collection of 42 *L. monocytogenes* strains. All strains were inhibited but had different sensitivities. The effect of the initial concentration of the protective and the target bacteria revealed that the inhibition always occurred when *L. piscium* had reached its maximum population density, whatever the initial concentration of the protective bacteria. Viewed by scanning electron microscopy, *L. monocytogenes* cell shape and surface appeared modified in co-culture with *L. piscium* CNCM I-4031. Lastly, *L. monocytogenes* virulence, evaluated by a plaque-forming assay on the HT-29 cell line, was reduced after cell pre-treatment by the protective bacteria. In conclusion, the bioprotective effect of *L. piscium* toward *L. monocytogenes* growth and virulence was demonstrated, and a hypothesis for the inhibition mechanism is put forward.

## Introduction

*Listeria monocytogenes* is a human pathogenic Gram-positive bacterium, which is responsible for foodborne listeriosis generally associated with a high mortality rate (20–30%). All human population groups can be infected and particularly newborn infants, pregnant women, elderly people, and immuno-compromised patients (Lecuit et al., 2015). This species constitutes a major problem in refrigerated ready-to-eat (RTE) foods (Rocourt et al., 2003). According to the Codex Alimentarius Commission, RTE products are “any food which is normally eaten in its raw state or any food handled, processed, mixed, cooked, or otherwise prepared into a form which is normally eaten without further listericidal steps" (CAC, 2007). *L. monocytogenes* differs from most other food-borne pathogens in that it is ubiquitous and can grow or survive in most conditions encountered in the food chain and food-processing procedures (Buchanan et al., 2017). Among foodstuffs, fishery products recorded the highest level of non-compliance with EU safety criteria in 2015 (EFSA, 2016) while seafood products were implicated in 15% of *L. monocytogenes* outbreaks reported in Europe in the last 3 years. Its survival ability makes the control of this microorganism in food products, especially RTE foods, a major challenge. The lactic acid bacteria (LAB), generally recognized as safe (Salminen et al., 1998), are also present and dominant in RTE foods such as meat and seafood products stored under vacuum or modified atmosphere packaging. Selected LAB can limit the development of *L. monocytogenes* and are thus recognized as efficient bioprotective agents in food systems (Brillet et al., 2004; Nilsson et al., 2005; Tomé et al., 2006; Vermeiren et al., 2006a; Unlu et al., 2015). The competition between LAB and *L. monocytogenes* involves various bactericidal or bacteriostatic mechanisms such as (i) competition for nutrients (Nilsson et al., 2005); (ii) production of one or more antimicrobial active metabolites such as bacteriocins (Richard et al., 2003; Dortu et al., 2008; Martinez et al., 2015), reuterin (El-Ziney et al., 1999), organic acids (Amezquita and Brashears, 2002), and hydrogen peroxide (Ito et al., 2003; Batdorj et al., 2007). *Lactococcus piscium* CNCM I-4031 is an efficient bioprotective strain for seafood products isolated from raw salmon stored under modified atmosphere packaging (Matamoros et al., 2009a). It improves the sensory quality of cooked shrimp by preventing the growth of *Brochothrix thermosphacta* (Fall et al., 2012). *L. piscium* CNCM I-4031 can also limit the growth of *L. monocytogenes* RF191 during the storage of cooked shrimp (Fall et al., 2010). The inhibition mechanism has not yet been entirely elucidated but using a chemically defined medium (MSMA) to mimic the shrimp matrix, it has been suggested that cell contact is required for the inhibition of *L. monocytogenes* RF191 by *L. piscium* CNCM I-4031 (Saraoui et al., 2016).

The aim of this study was to characterize the further inhibitory effect of *L. piscium* CNCM I-4031 against *L. monocytogenes* species. First, the inhibitory activity of the CNCM I-4031 strain was evaluated on a collection of 42 *L. monocytogenes* strains. Then, the effect of the protective strain CNCM I-4031 on the growth, morphological shape, and virulence of *L. monocytogenes* was investigated.

## Materials and Methods

### Bacterial Strains, Culture Media, and Conditions

*Lactococcus piscium* CNCM-I 4031 was isolated from fresh salmon steak packed under modified atmosphere packaging (Matamoros et al., 2009b). The *L. monocytogenes* strains used in this study are listed in **Table 1**. All strains were stored in aliquots of 250 μl at −80°C in a final concentration of 10% (v/v) of glycerol. For all experiments, an aliquot of the strain was subcultured in Elliker broth (Biokar Diagnostic, Beauvais, France) for 24 h at 26°C for *L. piscium*, and in Brain Heart Infusion supplemented by 2% NaCl (mBHI) (Biokar Diagnostic, Beauvais, France) for 24 h at 20°C for *L. monocytogenes*. The cultures were diluted in their culture medium to obtain appropriate initial cell concentrations. The chemically defined medium MSMA used for the bacterial interaction observation was prepared as previously described by Saraoui et al. (2016). *L. piscium* was enumerated by spreading 100 μl of 10-fold serial dilutions on Elliker agar plates incubated at 8°C for 5 days under anaerobiosis (co-cultures) or at 26°C for 48 h (pure cultures). *L. monocytogenes* was enumerated by spread-plating 100 μl (classic enumeration) or 5 μl (microenumeration method) of 10-fold serial dilutions on mBHI agar incubated at 37°C for 24 h.

**Table 1:** List of *L. monocytogenes* strains used in this study.

Strain	Origin	Country	Collection
ScottA	Milk (incriminated in listeriosis)	United States	CIP 103575
EGD-e	Rabbit (incriminated in listeriosis)	England	ATCC BAA-679
EU2208	Cod croquette	Spain	AZTI
EU2169	Fresh trout	Spain	AZTI
EU2170	Fresh trout	Spain	AZTI
EU2171	Fresh trout	Spain	AZTI
EU2148	Shrimp	Iceland	MATIS
EU2209	Smoked cod	Spain	AZTI
EU2158	Smoked salmon production plant	France	ASEPT
EU2159	Smoked salmon production plant	France	ASEPT
EU2160	Smoked salmon production plant	France	ASEPT
EU2161	Smoked salmon production plant	France	ASEPT
EU2162	Smoked salmon production plant	France	ASEPT
EU2163	Smoked salmon production plant	France	ASEPT
EU2164	Smoked salmon production plant	France	ASEPT
RF101	Smoked salmon production plant	France	ASEPT
RF102	Smoked salmon production plant	France	ASEPT
RF103	Smoked salmon production plant	France	ASEPT
RF104	Smoked salmon production plant	France	ASEPT
RF105	Smoked salmon production plant	France	ASEPT
RF106	Smoked salmon production plant	France	ASEPT
RF96	Smoked salmon production plant	France	ASEPT
RF97	Smoked salmon production plant	France	ASEPT
RF98	Smoked salmon production plant	France	ASEPT
RF99	Smoked salmon production plant	France	ASEPT
RF113	Smoked salmon production plant	France	ASEPT
RF115	Smoked salmon production plant	France	ASEPT
RF116	Smoked salmon production plant	France	ASEPT
RF118	Smoked salmon production plant	France	ASEPT
RF120	Smoked salmon production plant	France	ASEPT
RF122	Smoked salmon production plant	France	ASEPT
RF123	Smoked salmon production plant	France	ASEPT
RF124	Smoked salmon production plant	France	ASEPT
RF125	Smoked salmon production plant	France	ASEPT
RF133	Smoked salmon production plant	France	ASEPT
RF135	Smoked salmon production plant	France	ASEPT
RF138	Smoked salmon production plant	France	ASEPT
RF142	Smoked salmon production plant	France	ASEPT
RF152	Smoked salmon production plant	France	ASEPT
RF92	Smoked trout	France	Aqualande
RF93	Smoked trout	France	Aqualande
RF166	Taramasalata	France	Biocéane
RF191	Tropical cooked peeled shrimp	France	PFI Nouvelles Vagues

*ATCC, American Type Culture Collection; AZTI, Derio, Spain; MATIS, Reykjavik, Iceland; ASEPT, Le Mans, France; Aqualande, Roquefort, France; Biocéane, Nantes, France; PFI Nouvelles Vagues, Boulogne-sur-Mer, France.*

### Biodiversity of *L. monocytogenes* Sensitivity to *L. piscium* CNCM I-4031

After subculture, *L. piscium* CNCM I-4031 and each of the 42 *L. monocytogenes* strains (**Table 1**) were co-cultured in a 96-well microplate filled with 200 μl of MSMA at an initial concentration of 10^6^ and 10^3^ CFU/ml, respectively. The microplate was incubated at 26°C for 30 h without shaking. Controls consisted of monocultures of each *L. monocytogenes* strain inoculated in the same conditions. *L. monocytogenes* strains were enumerated in CFU/ml using the microenumeration method described in section “Bacterial Strains, Culture Media, and Conditions.” The inhibition was calculated by the difference in the log-concentration of *L. monocytogenes* strain in pure culture and in co-culture. The standard deviation was estimated according to five independent replicates of the inhibition tests for *L. monocytogenes* RF191 and *L. piscium* CNCM-4031.

### Effect of Co-culture Ratios on the Inhibition of *L. monocytogenes* by *L. piscium* CNCM I-4031

*Lactococcus piscium* CNCM I-4031 and *L. monocytogenes* RF191 were co-inoculated in 250-ml flasks of MSMA medium, without shaking. Depending on the experiments, the initial ratios between *L. piscium* and *L. monocytogenes* were 10^3^/10^3^ (A), 10^5^/10^3^ (B), and 10^6^/10^6^ (C) CFU/ml, respectively. The co-cultures were incubated at 26°C, and the growth of both strains was monitored during 48 to 72 h by the classic enumeration method described in section “Bacterial Strains, Culture Media, and Conditions.” Controls consisted of monocultures of each strain in MSMA at 26°C at the same inoculation levels. All the cultures were performed in triplicate.

### Observation of Cells in Co-culture by Scanning Electron Microscopy

Pure cultures and co-cultures (ratio 10^6^/10^3^ CFU/ml) of *L. piscium* CNCM I-4031 and *L. monocytogenes* RF191 were cultivated in 10 ml of MSMA medium at 26°C for 24 h. Then, 1 ml (10^8^ cells) of the suspension was filtered on a Nuclepore^®^ polycarbonate membrane with a 0.22-μm pore size and 13-mm diameter (Whatman International Ltd., Maidstone, United Kingdom). In order to observe the bacterial interaction on a solid medium (Dubey and Ben-Yehuda, 2011), another filter membrane was placed on an MSMA agar (15 g/l) plate and spotted with 10 μl of co-culture and incubated for 6 h at 26°C. All membranes containing the cells were fixed with 2.5% (v/v) glutaraldehyde (diluted in sodium cacodylate 0.1 M, pH 7.2) (Sigma Aldrich, Saint-Quentin Fallavier, France) for 48 h at 4°C. The fixing solution was renewed twice. Then, the samples were washed using a solution of sodium cacodylate (0.2 M, pH 7.2) and dehydrated in serial concentrations of ethanol (60, 70, 80, 90, 95%), 10 min for each concentration, followed by three times/20 min in 100% ethanol. The membranes were transferred to a critical point dryer, and the samples were subsequently sputter-coated and observed with a scanning electron microscope (Jeol JSM 6301F) at the CMEBA platform (Rennes, France).

### Analysis of the Virulence of *L. monocytogenes* Co-cultivated With *L. piscium*

*Lactococcus piscium* CNCM I-4031, *L. monocytogenes* RF191, and *L. monocytogenes* Scott A strains were cultivated in MSMA medium at 26°C for 24 h in triplicates. The cultures were centrifuged, re-suspended in phosphate buffered-saline (PBS, Eurobio, Courtaboeuf, France), and then diluted to obtain appropriate cell concentrations for the cell line infection.

The human adenocarcinoma cells (line HT-29) (European Collection of Animal Cell Cultures, Salisbury, United Kingdom) were routinely grown in 75-cm^2^ flasks (Sigma) in a complete medium, DMEM (Dulbecco’s modified Eagle’s medium), with 10% (v/v) fetal calf serum (SCF), and 1% amphotericin B 250 μg/ml (Eurobio). Gentamicin 100 μg/ml (Sigma) was added to the culture medium. Cells were kept in the humidified atmosphere of a 5% CO_2_ incubator at 37°C. One hundred microliter of HT-29 cell suspension (2 × 10^4^ cells) was deposited per well in a 96-well tissue culture plate (Sigma). The plates were incubated for 4 days with antibiotics followed by incubation for 24 h without antibiotics to obtain confluent monolayers.

In each well, HT-29 cells were infected with 10^8^ *L. piscium* CNCM I-4031 and incubated for 1 h at 37°C. *L. piscium* cells were then removed by suction, and the HT-29 cells were infected with 100-μl suspensions from 10^8^ to 10^3^ CFU/ml of *L. monocytogenes* per well and incubated for 2 h at 37°C. *L. monocytogenes* cells were then removed by suction. The HT-29 cells were covered with 100 μl of DMEM 10% SCF with 100 μg/ml of gentamicin and incubated for 1.5 h at 37°C to eliminate bacterial cells from the plates. Each well was then overlaid with DMEM 10% SCF with 100 μg/ml of gentamicin containing 0.47% of indubiose to prevent cell starvation. Incubation was carried out at 37°C for 24 to 48 h. The virulence of the bacterial cells was evaluated by plaque-forming assay (PFA) in the cell monolayers using an optical microscope (VWR, Pennsylvania, United States). Controls consisted of HT-29 cells infected with serial dilutions of 10^8^ to 10^2^ UFC of *L. piscium* CNCM I-4031 or *L. monocytogenes* RF191 or ScottA per well. The whole experiment was repeated three times (different weeks) corresponding to nine independent tests.

### Statistical Analyses

Statistical analyses on bacterial counts concentrations were performed using R software (R Core Team, 2014) by the analysis of one-way analysis of factor variance (ANOVA) followed by least significant difference (LSD) test.

## Results and Discussion

### Inhibition Capacity of *L. piscium* CNCM I-4031 Strain Toward Various *L. monocytogenes* Strains

**FIGURE 1:**
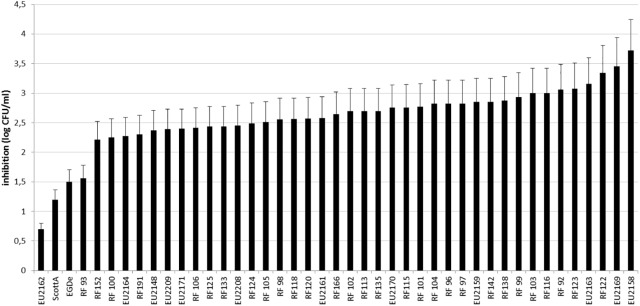
Inhibition of 42 *L. monocytogenes* strains after a 30-h culture with *L. piscium* CNCM I-4031 in MSMA.

**FIGURE 2:**
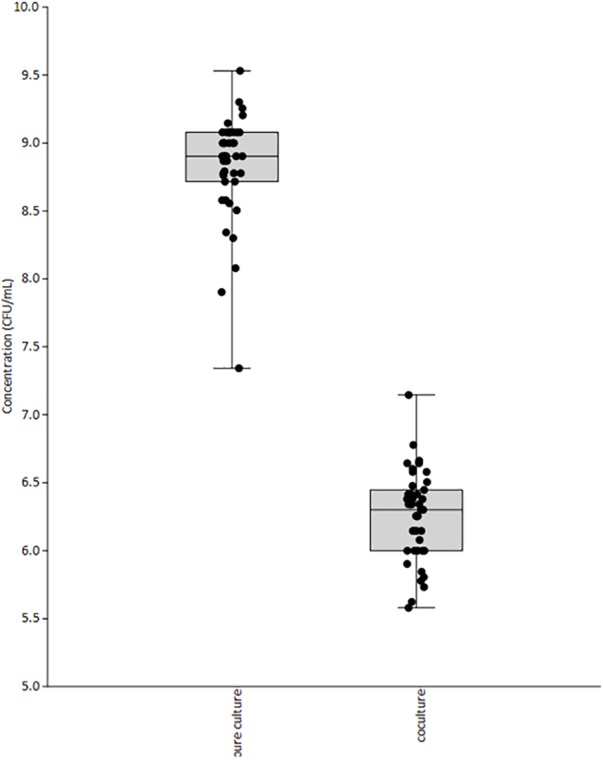
Final concentration of 42 *L. monocytogenes* strains in pure and mixed culture with *L. piscium* CNCM I-4031, after 30 h in MSMA medium.

A total of 42 *L. monocytogenes* strains from diverse sources, and geographical origins were selected (**Table 1**). Most of them were isolated from seafood except the reference strains ScottA (Fleming et al., 1985) and EGD-e (Murray et al., 1926) isolated during a listeriosis outbreak from human and animal tissue, respectively. The inhibition of *L. monocytogenes* strains was measured after 30 h of co-culture on microplates with *L. piscium* CNCM I-4031, in MSMA liquid medium. In these miniaturized experimental conditions, *L. monocytogenes* RF191 displayed an inhibition of 2.40 ± 0.35 log CFU/ml, which is lower than that previously described in larger culture volumes with an inhibition of 3–4 log CFU/ml (Saraoui et al., 2016). However, this screening test showed that *L. piscium* CNCM I-4031 could inhibit all *L. monocytogenes* strains, whatever their origin (**Figure 1**). The inhibition yield was strain-dependent, varying between 0.69 log CFU/ml for EU2162 and 3.72 log CFU/ml for EU2158. These values can be correlated with the low growth in MSMA medium of EU2262 in pure culture (7.3 log CFU/ml) and, in contrast, the strong growth of EU2158 (9.3 log CFU/ml). In mixed culture, after 30 h of incubation, the concentration of *L. monocytogenes* ranged between 5.6 and 7.1 log CFU/ml when cultivated alone and 7.3 and 9.5 in co-culture (**Figure 2**).

### Influence of the Initial Ratio of *L. piscium* CNCM I-4031/*L. monocytogenes*

**FIGURE 3:**
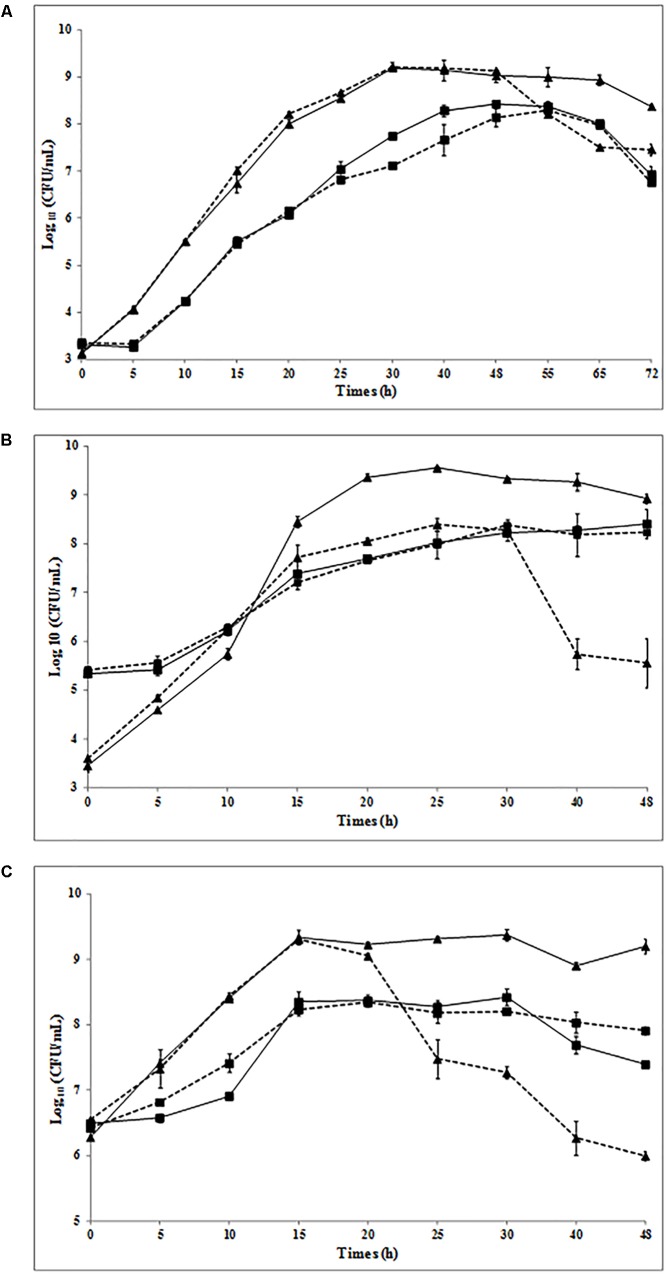
Growth of *L. piscium* CNCM I-4031 (■) and *L. monocytogenes* RF191 (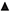) in pure culture (solid line) and in co-culture (dotted line) in MSMA at 26°C. Initial concentrations of *L. piscium/L. monocytogenes*: **(A)** 10^3/^10^3^ CFU/ml, **(B)** 10^5^/10^3^ CFU/ml, and **(C)** 10^6^/10^6^ CFU/ml.

Previous studies have shown that *L. piscium* CNCM I-4031 can inhibit the growth of *L. monocytogenes* RF191 from 3 to 4 log units with an inoculum ratio of 10^6^/10^3^ CFU/ml (*L. piscium/L. monocytogenes*) in shrimp at 8°C and in MSMA medium at 26°C (Fall et al., 2010; Saraoui et al., 2016). Considering that the concentration of pathogenic bacteria in food at the beginning of storage is low, protective bacteria are usually added at high concentrations to food products (Ananou et al., 2005; Brillet et al., 2005). In order to determine whether the inhibition was linked with these initial concentrations, three different initial ratios of *L. piscium*/*L. monocytogenes* RF191 were analyzed (**Figure 3**): 10^3^/10^3^ CFU/ml (A), 10^5^/10^3^ CFU/ml (B), and 10^6^/10^6^ CFU/ml (C).

The growth kinetics of *L. piscium* in pure and co-culture were similar whatever the initial ratios. The maximum population density (MPD) of approximately 10^8^ CFU/ml was reached after 40–48 h in (A), 25 h in (B), and 15 h in (C) with a growth rate between 0.32 to 0.45 h^−1^ (**Table 2**). *L. monocytogenes* growth started with an exponential phase and reached the MPD (∼10^9^ CFU/ml) at 30 h in (A), 20 h in (B), and 15 h in (C) (**Table 2**). In each co-culture, the *L. monocytogenes* population increased to reach 10^8^ to 10^9^ CFU/ml until *L. piscium* achieved the MPD, then decreased (**Figure 3**). These results indicate that, regardless of the inoculum ratio of the two strains, *L. monocytogenes* was always significantly inhibited in co-cultures when *L. piscium* reached its MPD. This inhibition was proportional to the initial concentration of *L. piscium* and ranged from 1.42 to 3.37 log CFU/g (**Table 2**) with a higher inhibitory effect when the protective strain was inoculated at 10^5^–10^6^ CFU/ml. These observations are in accordance with previous studies with other bioprotective LAB isolated from seafood, such as a non-bacteriocin-producing *Carnobacterium piscicola* A9b (Nilsson et al., 2005) and *Lactobacillus sakei* 10A (Vermeiren et al., 2006b). Nevertheless, contrary to *L. sakei* 10A, which required an initial concentration of up to 10^5^ CFU/g to inhibit *L. monocytogenes* effectively, *L. piscium* showed an inhibition with a low initial concentration of 10^3^ CFU/ml.

**Table 2:** Maximal population density and growth rate of *L. piscium* CNCM I-4031 (Lp) and *L. monocytogenes* RF191 (Lm) in pure (PC) or co-culture (CC) in MSMA medium at 26°C.

	*Lactococcus piscium* CNCM I-4031						*Listeria monocytogenes* RF191					
Initial ratio UFC/ml (Lp/Lm)	(A) 10^3^/10^3^		(B) 10^5^/10^3^		(C) 10^6^/10^6^		(A) 10^3^/10^3^		(B) 10^5^/10^3^		(C) 10^6^/10^6^	
Culture specifications	PC	CC	PC	CC	PC	CC	PC	CC	PC	CC	PC	CC
Maximal population density (log CFU/ml) ± SD	8.28 ± 0.12	8.14 ± 0.2	8.01 ± 0.01	7.98 ± 0.27	8.35 ± 0.15	8.23 ± 0.09	9.19 ± 0.04	9.21 ± 0.09	9.36 ± 0.03	8.05 ± 0.07	9.33 ± 0.11	9.31 ± 0.05
Growth rate (h^-1^)	0.43	0.40	0.45	0.37	0.40	0.32	0.60	0.63	0.76	0.66	0.51	0.63
Final population density (log CFU/ml) ± SD	6.92 ± 0.02^a^	6.75 ± 0.02^a^	8.40 ± 0.29^a^	8.23 ± 0.03^a^	7.39 ± 0.04^b^	7.91 ± 0.05^a^	8.37 ± 0.11^a^	7.46 ± 0.04^b^	8.92 ± 0.09^a^	5.55 ± 0.50^b^	9.20 ± 0.10^a^	5.99 ± 0.06^b^

*SD, standard deviation. ^a,b^Represent groups determined with LSD test, a same letter indicates values not significantly different (p-value < 0.05 by one-way ANOVA) through one condition.*

The competition between LAB species and other populations in food and mixed cultures by one single “dominant” strain and when LAB have reached their maximum level is described in the literature as the “Jameson effect” (Jameson, 1962; Gimenez and Dalgaard, 2004). The Jameson effect is considered a race between species in order to maximize their growth by exploiting the environmental nutrients. Thus, the species that first reaches its MPD inhibits the growth of the other species (Mellefont et al., 2008; Cornu et al., 2011). However, in our experiments, we showed that the inhibition of *L. monocytogenes* by *L. piscium* occurred even when the concentration of the pathogenic bacteria was higher than that of the protective bacteria (**Figures 3A,C**). In these conditions, *L. monocytogenes* reached its MPD before *L. piscium* but no inhibition of *L. piscium* occurred. The same results were found when *L. monocytogenes* ScottA was co-inoculated with *Escherichia coli* whereas in co-culture with *Lactobacillus plantarum* or *Pseudomonas fluorescens*, the first strain reaching its MPD stopped the growth of the other one (Mellefont et al., 2008).

In a previous study, we demonstrated that the amount of lactic acid produced by *L. piscium* CNCM I-4031 in co-culture conditions was not responsible for the inhibition (Saraoui et al., 2016). Moreover, the supplementation of the co-culture with nutrients did not restore the ability of *L. monocytogenes* to grow, and no inhibition was observed when both cultures were separated by a 0.45-μm membrane. The present study evidenced that *L. piscium* could limit the growth of *L. monocytogenes* when it reached its maximum density, whatever its initial level. Inhibition phenomena linked to maximum cellular concentration have been shown in other LAB, and some authors have suggested that they could involve quorum sensing (Kuipers et al., 2000; Risøen et al., 2000; Rohde and Quadri, 2006; Moslehi-Jenabian et al., 2011; Rizzello et al., 2014). In our case, the mechanism remains unknown and has still to be investigated.

### Observation of Cells in Co-culture by Scanning Electron Microscopy

**FIGURE 4:**
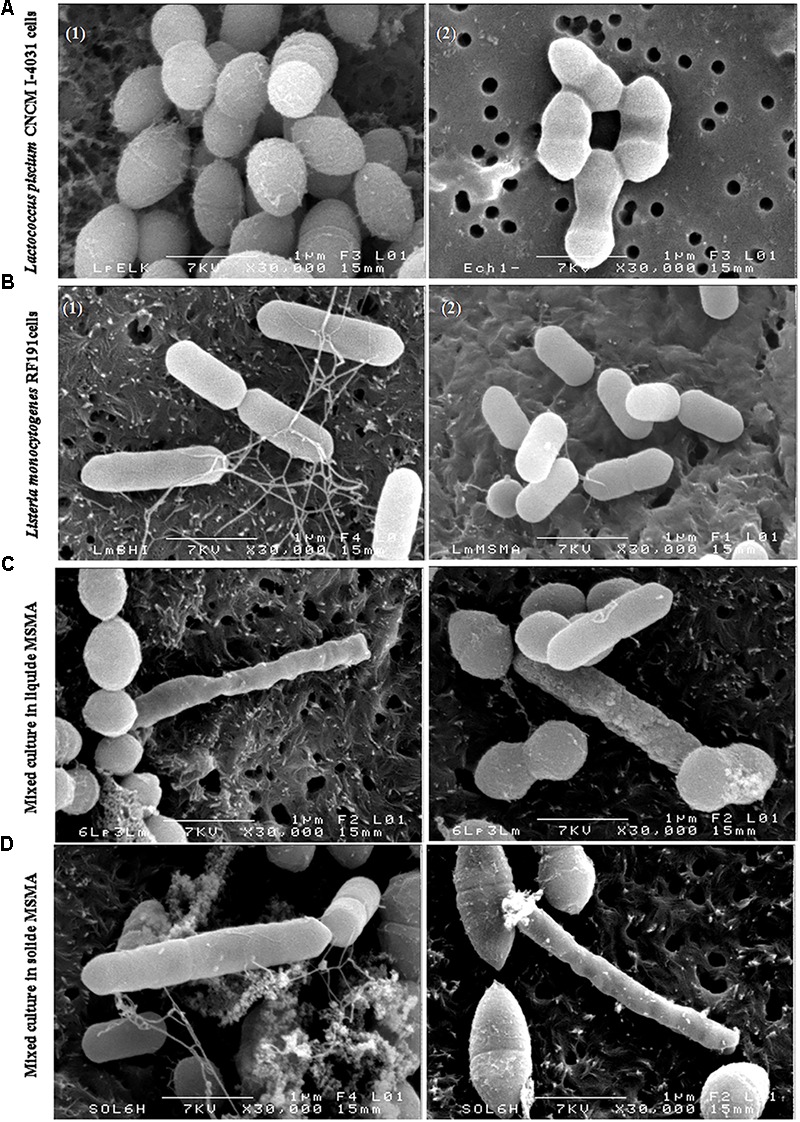
*L. piscium* CNCM I-4031 and *L. monocytogenes* RF191 cells in pure and co-culture viewed using scanning electron microscopy on polycarbonate membranes. Magnification: × 30,000. **(A)** *L. piscium* in pure culture (1) in Elliker; (2) in MSMA, 24 h at 26°C. **(B)** *L. monocytogenes* RF191 in pure culture (1) in mBHI; (2) in MSMA, 24 h at 26°C. **(C)** Co-culture of *L. piscium* CNCM I-4031 and *L. monocytogenes* RF191 in liquid MSMA. **(D)** Co-culture of *L. piscium* CNCM I-4031 and *L. monocytogenes* RF191 on MSMA plate.

With the aim of investigating the behavior of the pathogenic bacteria in co-culture at the microscopic scale, the cell morphology of *L. piscium* CNCM I-4031 and *L. monocytogenes* RF191 was compared in monoculture in MSMA medium with co-culture conditions at the time of inhibition. The results presented in **Figure 4** show that *L. piscium* in mono-cultures were spherical (Elliker) or ovoid (MSMA) cells between 0.5 and 1 μm in diameter and appeared in pairs or short chains. *L. monocytogenes* were rod-shaped cells between 0.5 μm (MSMA) and 1.5 μm (mBHI) in length and ∼0.5 μm in width, appearing individually or in pairs (**Figure 4B**). For co-cultures, strains were observed in either liquid or solid media (**Figures 4C,D**). Co-cultures on solid media have already revealed the presence of nanotubes, which are extensions of the cytoplasmic membrane used by bacteria to exchange their cellular compounds in cell contact interactions (Dubey and Ben-Yehuda, 2011). These authors described intra-species nanotubes between cells of *Bacillus subtilis*, as well as inter-species connections between cells of *B. subtilis*, *S. aureus*, and *E. coli* using scanning electron microscopy. In the conditions of our experiments, such nanotubes were not observed between the cells. However, the pictures show that in the presence of *L. piscium*, *L. monocytogenes* cells appeared to be more elongated (**Figures 4C,D**). The elongation of *Listeria* cells in stress conditions (low pH, high salt concentration) has been reported in previous studies (Besnard et al., 2000; Bereksi et al., 2002). *Listeria* cells can divide without septation, leading to the modification of their surface properties with the presence of filamentous structures. Our observations also suggest that the surface of *L. monocytogenes* cells was damaged or completely altered in the presence of LAB (**Figures 4C,D**). These effects on *L. monocytogenes* morphology have been reported in previous studies dealing with the mechanism of action of anti-*Listeria* components (Dieuleveux et al., 1998).

### Effect of *L. piscium* CNCM I-4031 on the Virulence of *L. monocytogenes*

**FIGURE 5:**
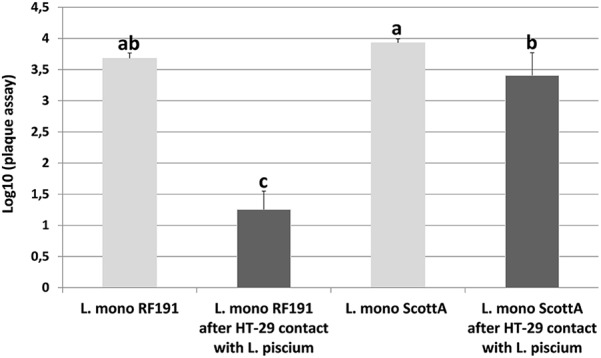
*L. monocytogenes* plaque-forming assay (PFA) using HT-29 epithelial cells infected with strains ScottA and RF191 directly or after 1 h of pre-incubation with *L. piscium* CNCM I-4031. Plaques were counted in HT-29 cells infected with 10^5^ UFC/ml of *L. monocytogenes*. No plaque was observed when *L. piscium* CNCM I-4031 was inoculated alone. Error bars indicate standard deviations from at least three independent experiments. ^a,b,c^Represent groups determined with LSD test, a same letter indicates values not significantly different (*p*-value < 0.05 by one way-ANOVA).

As an important pathogen, *L. monocytogenes* encompasses a large spectrum of strains with varying virulence effects (Buchanan et al., 2017). In order to examine another aspect of the bioprotective effect of *L. piscium*, the impact on *L. monocytogenes* virulence was investigated. For this purpose, the virulence of two selected *L. monocytogenes* strains, RF191 isolated from seafood and ScottA known to be a highly virulent strain (Lindback et al., 2010), were tested using an HT-29 cell PFA, in the presence or absence of *L. piscium*. On an HT-29 monolayer pre-treated with 10^8^ *L. piscium*, no lysis plates were detected after 24 or 48 h confirming that *L. piscium* has no pathogenic activity. Plaques were counted in the well containing HT-29 cells infected by 5 log CFU/ml of *L. monocytogenes* ScottA or RF191 strains. The ScottA strain formed large deep plaques whereas the RF191 strain formed small shallow ones. The mean log PFA values were 3.93 ± 0.06 and 3.68 ± 0.08, respectively (**Figure 5**). According to the study of Roche et al. (2001), the RF191 strain that forms more than 3.34 log plaques should be considered virulent, even though it remains less virulent than the ScottA strain. When HT-29 cells were infected by *L. monocytogenes* strains after being treated by *L. piscium*, the mean log PFA values were 3.40 ± 0.36 and 1.25 ± 0.29 for ScottA and RF191 strains, respectively (**Figure 5**). A significant effect was found for the RF191 strain that had lost its virulence after pre-treatment with *L. piscium* CNCM I-64031. A slight but a significant decrease in virulence was also observed for the ScottA strain, suggesting that the effect of the protective bacteria on *L. monocytogenes* virulence is strain-dependent. The effect on virulence reduction by LAB has already been described for foodborne pathogens such as *L. monocytogenes* (Garriga et al., 2015; Pilchova et al., 2016) or *Campylobacter* (Alemka et al., 2010). The effect is usually investigated as one of the probiotic properties of bacteria; however, such additional properties also increase the bioprotective value of strains and their safety assessment.

## Conclusion

In this study, we have demonstrated the ability of *L. piscium* CNCM I-4031 to inhibit a large collection of *L. monocytogenes* strains. This inhibition occurs whatever the initial concentration of the protective strain when *L. piscium* has reached its MPD. The inhibition mechanism is still under investigation but our different sets of results suggest that it requires the proximity of cells and affects the cellular surface of the targeted bacteria. In addition to inhibiting *L. monocytogenes* growth, *L. piscium* decreased *L. monocytogenes* virulence with a variable effect according to the strain. This study provides additional knowledge about the inhibitory activity of a non-bacteriocin-producing LAB toward *L. monocytogenes* and significant information for the potential use of *L. piscium* CNCM I-4031 to control *L. monocytogenes* in seafood products.

## Author Contributions

M-FP and FL designed the work. TS, DP, and FC performed the experiments. TS and FL assisted with the scanning electron microscopy analysis. TS, M-FP, FL, DP, and J-MC analyzed the data. TS drafted the paper. MF-P, FL, and DP wrote the final version of the manuscript, which was read and approved by all authors.

## Conflict of Interest Statement

The authors declare that the research was conducted in the absence of any commercial or financial relationships that could be construed as a potential conflict of interest.

## References

[B1] Alemka A. Clyne M. Shanahan F. Tompkins T. Corcionivoschi N. Bourke B. (2010). Probiotic colonization of the adherent mucus layer of HT29MTXE12 cells attenuates Campylobacter jejuni virulence properties. *Infect. Immun.* 78 2812–2822. 10.1128/IAI.01249-09 20308300 PMC2876579

[B2] Amezquita A. Brashears M. M. (2002). Competitive inhibition of *Listeria monocytogenes* in ready-to-eat meat products by lactic acid bacteria. *J. Food Prot.* 65 316–325. 10.4315/0362-028X-65.2.316 11848562

[B3] Ananou S. Garriga M. Hugas M. Maqueda M. Martinez-Bueno M. Galvez A. (2005). Control of *Listeria monocytogenes* in model sausages by enterocin AS-48. Int. *J. Food Microbiol.* 103 179–190. 10.1016/j.ijfoodmicro.2004.12.024 16083820

[B4] Batdorj B. Trinetta V. Dalgalarrondo M. Prevost H. Dousset X. Ivanova I. (2007). Isolation, taxonomic identification and hydrogen peroxide production by *Lactobacillus delbrueckii* subsp. *lactis* T 31, isolated from Mongolian yoghurt: inhibitory activity on food-borne pathogens. *J. Appl. Microbiol.* 103 584–593. 10.1111/j.1365-2672.2007.03279.x 17714391

[B5] Bereksi N. Gavini F. Benezech T. Faille C. (2002). Growth, morphology and surface properties of *Listeria monocytogenes* Scott A and LO28 under saline and acid environments. *J. Appl. Microbiol.* 92 556–565. 10.1046/j.1365-2672.2002.01564.x 11872133

[B6] Besnard V. Federighi M. Cappelier J. M. (2000). Development of a direct viable count procedure for the investigation of VBNC state in *Listeria monocytogenes*. *Lett. Appl. Microbiol.* 31 77–81. 10.1046/j.1472-765x.2000.00771.x 10886620

[B7] Brillet A. Pilet M. F. Prévost H. Bouttefroy A. Leroi F. (2004). Biodiversity of *Listeria monocytogenes* sensitivity to bacteriocin-producing *Carnobacterium* strains and application in sterile cold-smoked salmon. *J. Appl. Bacteriol.* 97 1029–1037. 10.1111/j.1365-2672.2004.02383.x 15479419

[B8] Brillet A. Pilet M. F. Prévost H. Cardinal M. Leroi F. (2005). Effect of inoculation of *Carnobacterium divergens* V 41, a biopreservative strain against *Listeria monocytogenes* risk, on the microbiological, and sensory quality of cold-smoked salmon. *Int. J. Food Microbiol.* 104 309–324. 10.1016/j.ijfoodmicro.2005.03.012 15979753

[B9] Buchanan R. L. Gorris L. G. M. Hayman M. M. Jackson T. C. Whiting R. C. (2017). A review of *Listeria monocytogenes*: an update on outbreaks, virulence, dose-response, ecology, and risk assessments. *Food Control* 75 1–13. 10.1016/j.foodcont.2016.12.016

[B10] CAC (2007). *CAC/GL 61-2007. Guidelines on the Application of General Principles of Food Hygiene to the Control of Listeria monocytogenes in Ready-to-Eat Foods.* Geneva: Codex Alimentarius Commission, 1–28.

[B11] Cornu M. Billoir E. Bergis H. Beaufort A. Zuliani V. (2011). Modeling microbial competition in food: application to the behavior of *Listeria monocytogenes* and lactic acid flora in pork meat products. *Food Microbiol.* 28 639–647. 10.1016/j.fm.2010.08.007 21511123

[B12] Dieuleveux V. Lemarinier S. Gueguen M. (1998). Antimicrobial spectrum and target site of D-3-phenyllactic acid. *Int. J. Food Microbiol.* 40 177–183. 10.1016/S0168-1605(98)00031-2 9620125

[B13] Dortu C. Huch M. Holzapfel W. H. Franz C. M. Thonart P. (2008). Anti-listerial activity of bacteriocin-producing Lactobacillus curvatus CWBI-B28 and Lactobacillus sakei CWBI-B1365 on raw beef and poultry meat. *Lett. Appl. Microbiol.* 47 581–586. 10.1111/j.1472-765X.2008.02468.x 19120930

[B14] Dubey G. P. Ben-Yehuda S. (2011). Intercellular nanotubes mediate bacterial communication. *Cell* 144 590–600. 10.1016/j.cell.2011.01.015 21335240

[B15] EFSA (2016). The European Union summary report on trends and sources of zoonoses, zoonotic agents and food-borne outbreaks in 2015. *EFSA J.* 14:4364. 32625371 10.2903/j.efsa.2017.5077 PMC7009962

[B16] El-Ziney M. G. van den Tempel T. Debevere J. Jakobsen M. (1999). Application of reuterin produced by Lactobacillus reuteri 12002 for meat decontamination and preservation. *J. Food Prot.* 62 257–261. 10.4315/0362-028X-62.3.257 10090245

[B17] Fall P. A. Leroi F. Chevalier F. Guerin C. Pilet M. F. (2010). Protective effect of a non-bacteriocinogenic *Lactococcus piscium* CNCM I-4031 strain against *Listeria monocytogenes* in sterilized tropical cooked peeled shrimp. *J. Aquat. Food Prod. Technol.* 19 84–92. 10.1080/10498850.2010.486910

[B18] Fall P. A. Pilet M. F. Leduc F. Cardinal M. Duflos G. Guérin C. (2012). Sensory and physicochemical evolution of tropical cooked peeled shrimp inoculated by *Brochothrix thermosphacta* and *Lactococcus piscium* CNCM I-4031 during storage at 8°C. *Int. J. Food Microbiol.* 152 82–90. 10.1016/j.ijfoodmicro.2011.07.015 21835482

[B19] Fleming D. W. Cochi S. L. MacDonald K. L. Brondum J. Hayes P. S. Plikaytis B. D. (1985). Pasteurized milk as a vehicle of infection in an outbreak of listeriosis. *N. Engl. J. Med.* 312 404–407. 10.1056/NEJM198502143120704 3918263

[B20] Garriga M. Rubio R. Aymerich T. Ruas-Madiedo P. (2015). Potentially probiotic and bioprotective lactic acid bacteria starter cultures antagonise the *Listeria monocytogenes* adhesion to HT29 colonocyte-like cells. *Benef. Microbes* 6 337–343. 10.3920/BM2014.0056 25488261

[B21] Gimenez B. Dalgaard P. (2004). Modelling and predicting the simultaneous growth of *Listeria monocytogenes* and spoilage micro-organisms in cold-smoked salmon. *J. Appl. Microbiol.* 96 96–109. 10.1046/j.1365-2672.2003.02137.x 14678163

[B22] Ito A. Sato Y. Kudo S. Sato S. Nakajima H. Toba T. (2003). The screening of hydrogen peroxide-producing lactic acid bacteria and their application to inactivating psychrotrophic food-borne pathogens. *Curr. Microbiol.* 47 231–236. 10.1007/s00284-002-3993-1 14570275

[B23] Jameson J. E. (1962). A discussion of dynamics of *Salmonella* enrichment. *J. Hyg.* 60 193–207. 10.1017/S0022172400039462 14451045 PMC2134411

[B24] Kuipers O. P. Buist G. Kok J. (2000). Current strategies for improving food bacteria. *Res. Microbiol.* 151 815–822. 10.1016/S0923-2508(00)01147-5 11191806

[B25] Lecuit M. Charlier-Woerther C. Leclerq A. (2015). *Rapport Annuel D’activité du Centre national de Référence des Listeria – Année 2014.* Paris: Institut Pasteur.

[B26] Lindback T. Rottenberg M. E. Roche S. M. Rorvik L. M. (2010). The ability to enter into an avirulent viable but non-culturable (VBNC) form is widespread among *Listeria monocytogenes* isolates from salmon, patients and environment. *Vet. Res.* 41:8. 10.1051/vetres/2009056 19796607 PMC2775167

[B27] Martinez R. C. Staliano C. D. Vieira A. D. Villarreal M. L. Todorov S. D. Saad S. M. (2015). Bacteriocin production and inhibition of *Listeria monocytogenes* by *Lactobacillus sakei* subsp. *sakei* 2a in a potentially synbiotic cheese spread. *Food Microbiol.* 48 143–152. 10.1016/j.fm.2014.12.010 25791002

[B28] Matamoros S. Leroi F. Cardinal M. Gigout F. Kasbi Chadli F. Cornet J. (2009a). Psychrotrophic lactic acid bacteria used to improve the safety and quality of vacuum-packaged cooked and peeled tropical shrimp and cold-smoked salmon. *J. Food Prot.* 72 365–374. 19350982 10.4315/0362-028x-72.2.365

[B29] Matamoros S. Pilet M. F. Gigout F. Prévost H. Leroi F. (2009b). Selection and evaluation of seafood-borne psychrotrophic lactic acid bacteria as inhibitors of pathogenic and spoilage bacteria. *Food Microbiol.* 26 638–644. 10.1016/j.fm.2009.04.011 19527840

[B30] Mellefont L. A. McMeekin T. A. Ross T. (2008). Effect of relative inoculum concentration on *Listeria monocytogenes* growth in co-culture. *Int. J. Food Microbiol.* 121 157–168. 10.1016/j.ijfoodmicro.2007.10.010 18083261

[B31] Moslehi-Jenabian S. Vogensen F. K. Jespersen L. (2011). The quorum sensing luxS gene is induced in Lactobacillus acidophilus NCFM in response to *Listeria monocytogenes*. *Int. J. Food Microbiol.* 149 269–273. 10.1016/j.ijfoodmicro.2011.06.011 21784546

[B32] Murray E. G. D. Webb R. A. Swann M. B. R. (1926). A disease of rabbits characterised by a large mononuclear leucocytosis, caused by a hitherto undescribed bacillus Bacterium monocytogenes (n.sp.). *J. Pathol. Bacteriol.* 29 407–439. 10.1002/path.1700290409

[B33] Nilsson L. Hansen T. B. Garrido P. Buchrieser C. Glaser P. Knochel S. (2005). Growth inhibition of *Listeria monocytogenes* by a non bacteriocinogenic *Carnobacterium piscicola*. *J. Appl. Microbiol.* 98 172–183. 10.1111/j.1365-2672.2004.02438.x 15610430

[B34] Pilchova T. Pilet M. F. Cappelier J. M. Pazlarova J. Tresse O. (2016). Protective effect of *Carnobacterium* spp. against *Listeria monocytogenes* during host cell invasion using in vitro HT29 model. *Front. Cell. Infect. Microbiol.* 6:88. 10.3389/fcimb.2016.00088 27617232 PMC4999452

[B35] R Core Team (2014). *A Language and Environment for Statistical Computing [Internet].* Vienna: R Foundation for Statistical Computing.

[B36] Richard C. Brillet A. Pilet M. F. Prévost H. Drider D. (2003). Evidence on inhibition of *Listeria monocytogenes* by divercin V41 action. *Lett. Appl. Microbiol.* 36 288–292. 10.1046/j.1472-765X.2003.01310.x 12680940

[B37] Risøen P. A. Brurberg M. B. Eijsink V. G. H. Nes I. F. (2000). Functional analysis of promoters involved in quorum sensing-based regulation of bacteriocin production in Lactobacillus. *Mol. Microbiol.* 37 619–628. 10.1046/j.1365-2958.2000.02029.x 10931355

[B38] Rizzello C. G. Filannino P. Di Cagno R. Calasso M. Gobbetti M. (2014). Quorum-sensing regulation of constitutive plantaricin by *Lactobacillus plantarum* strains under a model system for vegetables and fruits. *Appl. Environ. Microbiol.* 80 777–787. 10.1128/AEM.03224-13 24242246 PMC3911083

[B39] Roche S. M. Velge P. Bottreau E. Durier C. Marquet-van der Mee N. Pardon P. (2001). Assessment of the virulence of *Listeria monocytogenes*: agreement between a plaque-forming assay with HT-29 cells and infection of immucompetent mice. *Int. J. Food Microbiol.* 68 33–44. 10.1016/S0168-1605(01)00460-3 11545218

[B40] Rocourt J. BenEmbarek P. Toyofuku H. Schlundt J. (2003). Quantitative risk assessment of *Listeria monocytogenes* in ready to eat foods: the FAO/WHO approach. *FEMS Immunol. Med. Microbiol.* 35 263–267. 10.1016/S0928-8244(02)00468-6 12648845

[B41] Rohde B. H. Quadri L. E. (2006). Functional characterization of a three-component regulatory system involved in quorum sensing-based regulation of peptide antibiotic production in *Carnobacterium maltaromaticum*. *BMC Microbiol.* 6:93. 10.1186/1471-2180-6-93 17054797 PMC1634752

[B42] Salminen S. von Wright A. Morelli L. Marteau P. Brassart D. de Vos W. M. (1998). Demonstration of safety of probiotics - a review. *Int. J. Food Microbiol.* 44 93–106. 10.1016/S0168-1605(98)00128-7 9849787

[B43] Saraoui T. Fall P. A. Leroi F. Antignac J.-P. Chéreau S. Pilet M. F. (2016). Inhibition mechanism of *Listeria monocytogenes* by a bioprotective bacteria *Lactococcus piscium* CNCM I-4031. *Food Microbiol.* 53 70–78. 10.1016/j.fm.2015.01.002 26611171

[B44] Tomé E. Teixeira P. Gibbs P. A. (2006). Anti-listerial inhibitory lactic acid bacteria isolated from commercial cold smoked salmon. *Food Microbiol.* 23 399–405. 10.1016/j.fm.2005.05.004 16943030

[B45] Unlu G. Nielsen B. Ionita C. (2015). Production of antilisterial bacteriocins from lactic acid bacteria in dairy-based media: a comparative study. *Probiotics Antimicrob. Proteins* 7 259–274. 10.1007/s12602-015-9200-z 26341641

[B46] Vermeiren L. Devlieghere F. Vandekinderen I. Debevere J. (2006a). The interaction of the non-bacteriocinogenic *Lactobacillus sakei* 10A and lactocin S producing *Lactobacillus sakei* 148 towards *Listeria monocytogenes* on a model cooked ham. *Food Microbiol.* 23 511–518. 16943045 10.1016/j.fm.2005.10.005

[B47] Vermeiren L. Devlieghere F. Vandekinderen I. Rajtak U. Debevere J. (2006b). The sensory acceptability of cooked meat products treated with a protective culture depends on glucose content and buffering capacity: a case study with *Lactobacillus sakei* 10A. *Meat Sci.* 74 532–545. 10.1016/j.meatsci.2006.05.003 22063058

